# Midgut Malrotation Causing Intermittent Intestinal Obstruction in a Young Adult

**DOI:** 10.1155/2014/758032

**Published:** 2014-05-20

**Authors:** Huseyin Kazim Bektasoglu, Ufuk Oguz Idiz, Mustafa Hasbahceci, Erkan Yardimci, Yurdakul Deniz Firat, Oguzhan Karatepe, Mahmut Muslumanoglu

**Affiliations:** General Surgery Department, Bezmialem Vakif University School of Medicine, Vatan Street, Fatih, 34093 Istanbul, Turkey

## Abstract

Midgut malrotation is a congenital anomaly of intestinal rotation and fixation that is generally seen in neonatal population. Adult cases are rarely reported. Early diagnosis is crucial to avoid life threatening complications. Here, we present an adulthood case of midgut volvulus as a rare cause of acute abdomen.

## 1. Introduction

Midgut malrotation (MM) as a congenital anomaly of intestinal rotation and fixation is generally seen in the neonatal population [1]. It usually occurs due to incomplete rotation or a complete fail of rotation in which the primitive intestinal loops ought to do around the superior mesenteric artery axis in the fetal period [2]. Rare adult cases have been reported [2–4]. Early diagnosis is crucial to avoid life-threatening complications such as volvulus, internal herniation, and intussusception [3]. While symptomatic malrotation is seen in one of 5000 live births, 90% of the infants with malrotation become symptomatic up to the end of the first year of life [5]. However, adult-age MM is difficult to diagnose and should be kept in mind in differential diagnosis of acute abdomen due to its nonspecific presentation. Here, we present a case of adult age chronic MM with recurrent abdominal pain attacks.

## 2. Case

**Figure 1:**
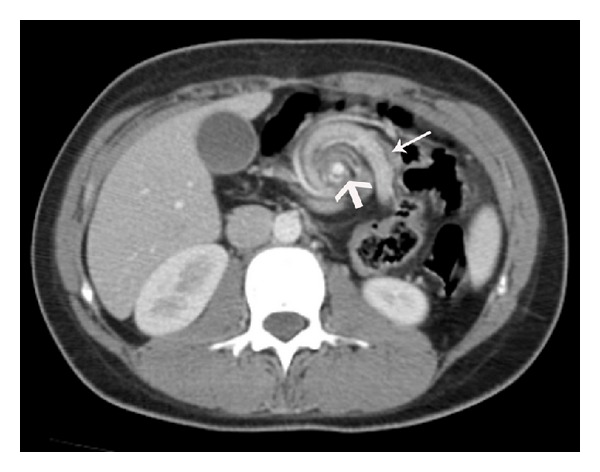
Axial contrast enhanced CT showing characteristic whirl-like appearance of superior mesenteric vein (thin arrow) wrapping around the superior mesenteric artery (thick arrow).

An 18-year-old female admitted to our emergency clinic with complaints of abdominal pain and vomiting for the last three days. Her past medical history revealed chronic abdominal pain and occasional vomiting since her childhood but she did not go under further investigation. Vital signs were within the normal range. Physical examination revealed only nonspecific abdominal tenderness without abdominal guarding, rebound tenderness, and abdominal distention. White blood cell count was 6500/*μ*L (normal range 4500–9900/*μ*L) and C-reactive protein value was 0.3 mg/L (normal range 0–5 mg/L). Computed tomography (CT) showed whirl-like appearance of the superior mesenteric vein and the bowel around the superior mesenteric artery axis (Figure 1). It was also noted that cecum and the ascending colon were predominately located on the left side, just adjacent to the sigmoid colon. The small intestines occupied right side of the abdomen.

**Figure 2:**
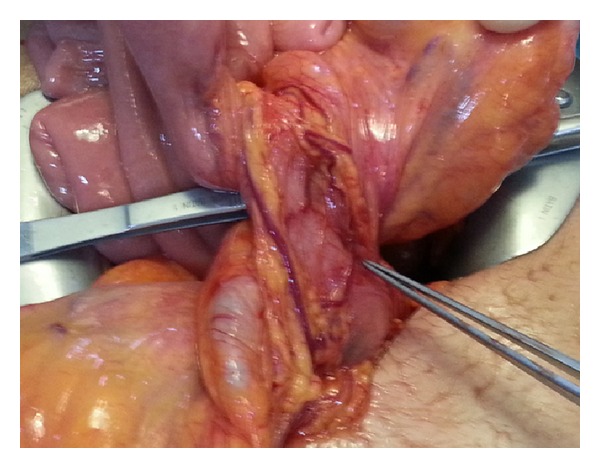
Rotation of the loops of small intestine along their vascular structures.

**Figure 3:**
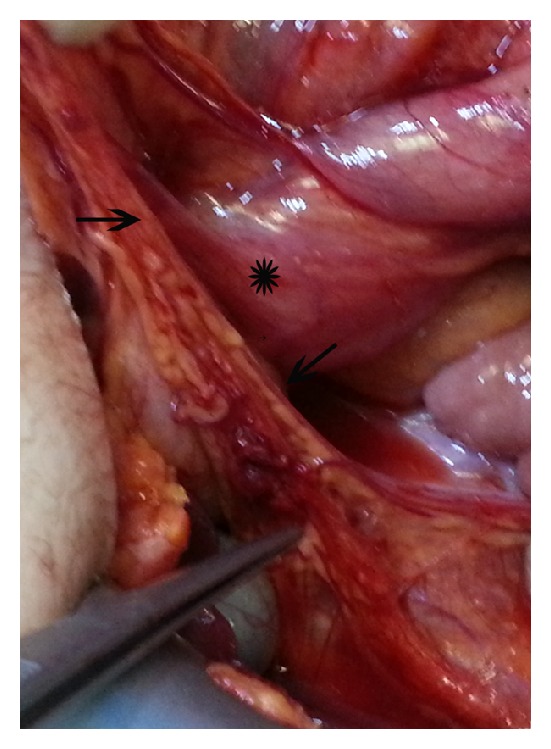
Ladd's bands (black arrows) impressing on the duodenum (asterisk).

**Figure 4:**
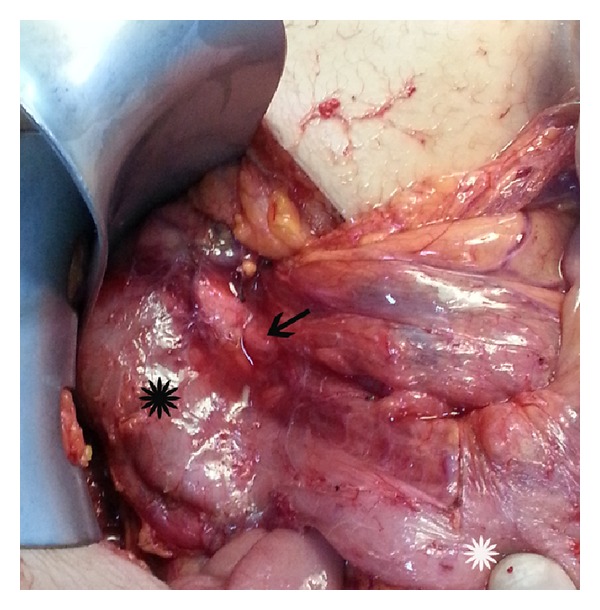
Right-sided duodenojejunal junction (white asterisk). The duodenum (black asterisk) and the head of the pancreas (black arrow) at their normal anatomic localizations.

**Figure 5:**
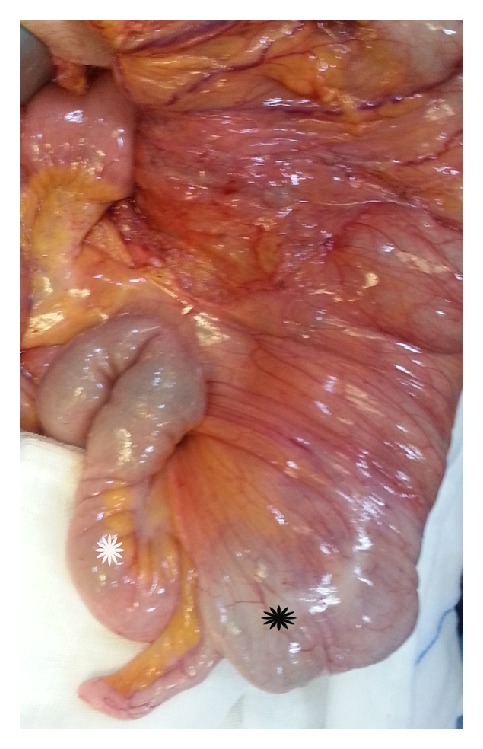
Left-sided cecum (black asterisk) and ascending colon and the terminal ileum (white asterisk) running to the right side.

The patient was taken to the operating theatre with the diagnosis of MM. At the exploration, rotation of minimally dilated small intestines (Figure 2) with no apparent intestinal ischemia was seen. Detorsion of the malrotated loops of the small intestine was performed in the anticlockwise direction. Intra-abdominal adhesions and Ladd's bands (Figure 3) as fibrous stalks of peritoneal tissues attaching cecum to the abdominal wall were dissected. The small intestines and the colon were placed to the right and the left side of the abdomen, respectively (Figures 4 and 5). Appendectomy was added. At the postoperative fourth day, she was discharged without any complaint. At the third month of the follow-up, she had no complaints with taking regular diet.

## 3. Discussion

Intestinal rotation and fixation begin at the 6th gestational week with a total of 270 degree anticlockwise rotation. At the 12th week, the intestinal segments were fixed as the ascending and descending colon on the right and left quadrants, respectively [6]. According to stringer classification which is based on the embryological state of development, there are three different types of MM as type I (nonrotation), type II (duodenal malrotation), and type III (combined duodenal and cecal malrotation) [7]. In this case, there was type I MM which includes left positioned cecum and ascending colon with right-sided duodenojejunal junction accompanied by inverted position of the superior mesenteric vessels and hypoplasia of the uncinate process of the pancreas.

Due to presence of the atypical symptoms, the diagnosis of MM is mostly made either incidentally during surgery or in autopsies [8]. There were many reports of MM cases that were operated due to ileus or acute appendicitis with lower left quadrant pain [9–11]. It is also possible to see an increased risk of morbidity and mortality due to delayed diagnosis of MM for various diseases such as acute appendicitis [12]. Although plain X-ray, Doppler ultrasound, and contrast studies might be used for diagnosis, it can be possible to diagnose this entity preoperatively by using CT scans due to the presence of specific anatomic features [13, 14]. In the present case, presence of intermittent attacks of intestinal obstruction in a young patient was regarded as a clue for the diagnosis of MM. Therefore, clinical suspicion and use of CT in selected cases can be regarded as the most appropriate way to reach the preoperative diagnosis [14].

In case of MM, the most commonly encountered presentation is the inability of cecum positioned to the right-lower quadrant and the pressure on the duodenum due to peritoneal Ladd's bands that fix the ectopically established cecum to the posterior wall [15]. Following description of the reduction of volvulus for the first time in 1932, Ladd described the liberalization of the bands that apply pressure to duodenum. Hence, the aim of Ladd's procedure is to dissect the bands that apply pressure to duodenum and jejunum and relief of the small intestine as in the present case [16].

As a conclusion, in case of intermittent intestinal obstruction in young adults, the physicians should keep in mind that early and accurate diagnosis of MM with an appropriate surgical treatment may save patients from unexpected complications. In acute presentations, CT should be chosen as the primary imaging modality.

## References

[1] Gamblin TC, Stephens RE, Johnson RK, Rothwell M (2003). Adult malrotation: a case report and review of the literature. *Current Surgery*.

[2] Zissin R, Rathaus V, Oscadchy A, Kots E, Gayer G, Shapiro-Feinberg M (1999). Intestinal malrotation as an incidental finding on CT in adults. *Abdominal Imaging*.

[3] Pickhardt PJ, Bhalla S (2002). Pictorial essay. Intestinal malrotation in adolescents and adults: spectrum of clinical and imaging features. *American Journal of Roentgenology*.

[4] Moldrem AW, Papaconstantinou H, Broker H, Megison S, Jeyarajah DR (2008). Late presentation of intestinal malrotation: an argument for elective repair. *World Journal of Surgery*.

[5] Ford EG, Senac MO, Srikanth MS, Weitzman JJ (1992). Malrotation of the intestine in children. *Annals of Surgery*.

[6] Kawahara R, Horiuchi H, Nogita H (2013). A case of cancer of the ampulla of Vater accompanied by malrotation. *The Kurume Medical Journal*.

[7] Ben Ely A, Gorelik N, Cohen-Sivan Y (2013). Appendicitis in adults with incidental midgut malrotation: CT findings. *Clinical Radiology*.

[8] Parish A (2002). Intestinal malrotation. *Medicine*.

[9] Goicochea Mancilla C, Díaz Plasencia J, Balmaceda Fraselle T (2001). Intestinal obstruction for malrotation in an adult patient. Report of a case. *Revista de Gastroenterologia del Peru*.

[10] Maxson RT, Franklin PA, Wagner CW (1995). Malrotation in the older child: surgical management, treatment, and outcome. *American Surgeon*.

[11] Van Roye S, Vandelanotte M, Proot L, Lanckneus M (1993). Chronic small bowel obstruction due to intestinal malrotation in the older child: an often missed diagnosis. *Acta Chirurgica Belgica*.

[12] Lin CJ, Tiu CM, Chou YH, Chen JD, Liang WY, Chang CY (2004). CT presentation of ruptured appendicitis in an adult with incomplete intestinal malrotation. *Emergency Radiology*.

[13] Camera L, Calabrese M, Mainenti PP (2012). Volvulus of the ascending colon in a non-rotated midgut: plain film and MDCT findings. *World Journal of Radiology*.

[14] Duran C, Öztürk E, Uraz S, Kocakuşak A, Mutlu H, Killi R (2008). Midgut volvulus: value of multidetector computed tomography in diagnosis. *Turkish Journal of Gastroenterology*.

[15] Karim MA, Mansour M, Ali A (2013). Re-do Roux-en-Y gastric bypass in a patient with known midgut malrotation. *Journal of the Society of Laparoendoscopic Surgeons*.

[16] Nakajima Y, Sakata H, Yamaguchi T (2013). Successful treatment of a 14-year-old patient with intestinal malrotation with laparoscopic Ladd procedure: case report and literature review. *World Journal of Emergency Surgery*.

